# Phenology-Dependent Sex Identification in Mature *Ginkgo biloba* Using Hyperspectral Imaging

**DOI:** 10.3390/plants15152255

**Published:** 2026-07-23

**Authors:** Zhengnan Zhao, Jingyue Zhang, Tao Wang, Si Liu, Zeyang Yi, Xue Wang, Xiao Chen, Qun Sun, Hongyan Sun

**Affiliations:** 1 Beijing Key Laboratory of Greening Plants Breeding, Beijing Academy of Forestry and Landscape Architecture, Beijing 100102, China; zhengnan2079@163.com (Z.Z.);; 2 College of Agronomy and Biotechnology, China Agricultural University, Beijing 100094, China; zhangjyxy6@163.com

**Keywords:** *Ginkgo biloba*, hyperspectral imaging, sex identification, phenological stage, flavonoid, cross-stage generalization

## Abstract

*Ginkgo biloba* is a dioecious species valued for landscaping and medicine, but rapid sex identification outside the flowering and fruiting stages remains challenging. Hyperspectral imaging offers a potential solution, though whether spectral sex markers are stable across phenological stages and can support a single year-round model is unclear. Here, leaf hyperspectral reflectance (400–1000 nm) was acquired from mature *G. biloba* at flowering (*n* = 360), green-leaf (*n* = 1395), and yellow-leaf (*n* = 243) stages. Sex identification models were built using multiple machine learning classifiers, with leaf flavonoid content as biochemical validation. Stage-specific models achieved optimal test accuracies of 96.67% (flowering, MSC + PLS-DA), 95.77% (green-leaf, raw spectra + LDA), and 97.12% (yellow-leaf, MSC + LDA). However, discriminative bands shifted from the visible (520–690 nm) at the green-leaf stage to the near-infrared (700–1000 nm) at the yellow-leaf stage, with almost no bands shared across all stages. Furthermore, cross-stage prediction accuracy dropped to near-chance levels (~50%). As t-SNE analysis revealed, the universal model had learned phenological rather than sex-specific information. In addition, flavonoid measurements revealed a highly significant sex × stage interaction (*p* < 0.001) and a reversal of the sex difference between green-leaf (male > female) and yellow-leaf (female > male) stages. Thus, the spectral and biochemical sex markers examined in this study varied substantially with phenology, and stage-specific models are required for practical sex identification in *G. biloba*.

## 1. Introduction

*Ginkgo biloba* L. is the oldest extant dioecious gymnosperm [1], valued for its ornamental, medicinal, and timber uses [2]. Male and female trees serve distinct purposes: male trees, with an upright form and no malodorous seeds, are preferred for urban landscaping, whereas female trees produce seeds prized for both culinary and medicinal properties [3]. Although mature trees can be sexed during flowering by the presence of male strobili or female ovules [4,5,6], the flowering period is largely confined to a short window in spring. After pollen dispersal, male trees revert to vegetative growth and no longer display conspicuous external sexual traits. Female trees, meanwhile, enter seed development after flowering, but the developing seeds lack distinct morphological features early on, making reliable identification difficult until autumn maturity. Existing sex identification techniques for *G. biloba* each have inherent limitations. Morphological observation during flowering is reliable but restricted to a brief seasonal window [4,5,6]. Electrochemical analysis can detect sex-related differences but is time-consuming and requires skilled operation [7]. Chromosome karyotyping offers high accuracy but is labor-intensive and destructive [8]. Molecular markers are highly specific and independent of phenology, yet they remain costly and operationally complex for large-scale field application [9]. Hence, developing a rapid and year-round applicable sexing method is of considerable practical value.

Year-round sex identification requires stable and detectable differences between male and female trees. A likely source of such differences lies in the well-documented influence of reproductive allocation on vegetative growth in dioecious plants [10,11]. Female individuals generally invest more resources in seed and fruit development [12], whereas males allocate resources to flower and pollen production [13]. This asymmetry in reproductive investment may lead to sexual divergence in physiological traits [14,15]. In poplars, such divergence has been shown to be phenology-dependent, as the sex-related difference in chlorophyll index shifts with phenological stage [16]. However, whether the physiological differences between male and female *G. biloba* trees likewise vary across phenological stages has yet to be systematically verified.

Hyperspectral imaging captures leaf reflectance across contiguous wavebands [17], and the resulting spectral signatures, reflecting pigment, water, and cellular structure composition [18,19], integrate the aforementioned physiological differences, thereby offering a non-destructive means of monitoring leaf physiological status. This technique is well suited to capturing the pronounced phenological changes that *G. biloba* leaves undergo over the annual cycle. Over this cycle, leaves transition from leaf expansion and active photosynthesis to senescence and yellowing, with pigment composition and photosynthetic capacity shifting markedly [20]. In addition, male and female trees exhibit inherent differences in leaf structural and hydraulic traits [21]. This physiological backdrop indicates that sexual divergence in *G. biloba* has a spectral basis detectable by hyperspectral imaging; however, the stability of these differences across phenological stages is a prerequisite for spectral sex markers to be applicable across stages.

A previous study adopted the latter approach, proposing a “stage-pre-discrimination” strategy that achieved satisfactory accuracy and first demonstrated the feasibility of hyperspectral imaging combined with machine learning for sexing mature *G. biloba* trees [22]. Whether a universal cross-stage model is equally feasible, however, remains an open question. If spectral sex markers are inherently stable across phenological stages, a universal model would streamline identification; if not, the stage-specific strategy becomes a biological necessity rather than merely a technical choice.

This study therefore investigates the stability of spectral sex markers across phenological stages through cross-stage transfer prediction, aiming to inform the choice of a hyperspectral sex identification strategy for *G. biloba*. To address this, leaf spectral data were acquired from mature trees at three phenological stages (flowering, green-leaf, and yellow-leaf) using visible and short-wave near-infrared (Vis–SW-NIR) hyperspectral imaging. Multiple machine learning methods were integrated to construct sex identification models, with leaf flavonoid content serving as a biochemical validation index. Specifically, this study (1) tests whether a year-round universal model is feasible by examining the cross-stage transferability of spectral sex markers, (2) characterizes the dynamic shifts in spectral differences and discriminative bands between the sexes across stages, and (3) links spectral features to flavonoid content to provide biochemical evidence for the observed spectral divergence.

## 2. Results

### 2.1. Hyperspectral Reflectance Curves of G. biloba Leaves

**Figure 1:**
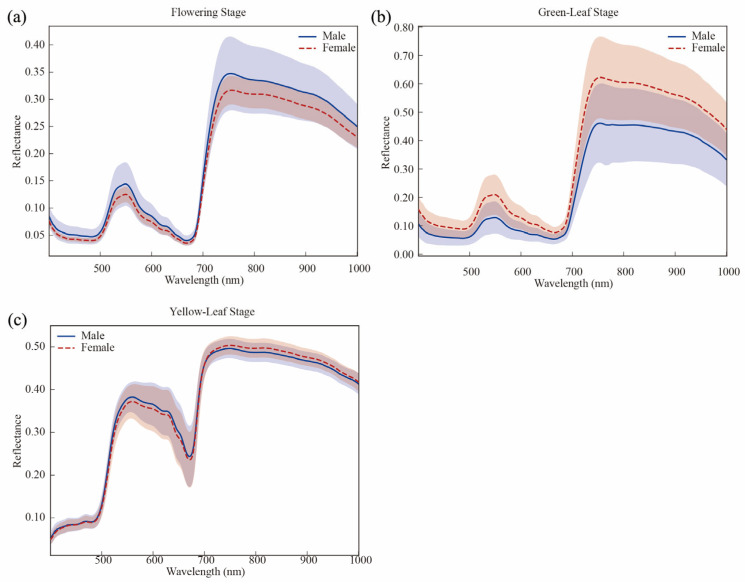
Mean hyperspectral reflectance curves of male and female *Ginkgo biloba* leaves at three phenological stages: (**a**) flowering, (**b**) green-leaf, (**c**) yellow-leaf. Solid and dashed lines represent the mean reflectance of male and female trees, respectively; shaded areas indicate standard deviation. Sample sizes: flowering, *n* = 360, green-leaf, *n* = 1395; yellow-leaf, *n* = 243.

The hyperspectral reflectance of mature *G. biloba* leaves exhibited marked phenological variation (Figure 1). In the visible region (400–700 nm), spectra at the flowering and green-leaf stages displayed typical plant reflectance features: a green peak near 550 nm and a red valley near 670 nm, corresponding to weak and strong chlorophyll absorption, respectively. At the yellow-leaf stage, as chlorophyll degraded and the carotenoid proportion increased, reflectance rose substantially between 500 and 700 nm; the red valley largely disappeared, and the green peak weakened. In the near-infrared (NIR) region (700–1000 nm), reflectance increased stepwise with developmental stage, from approximately 0.35 at flowering to 0.45–0.60 at the green-leaf stage, peaking around 0.50 at the yellow-leaf stage, reflecting phenological changes in leaf internal cellular structure.

Sex-related spectral differences also varied with phenology. At flowering, male trees showed slightly higher full-spectrum reflectance than females, although NIR standard deviations partially overlapped (Figure 1a). At the green-leaf stage, female trees exhibited markedly higher reflectance than males in both the visible and NIR regions, representing the most pronounced sex-based difference (Figure 1b). At the yellow-leaf stage, the spectral curves of the two sexes converged, and sex-related differences essentially disappeared (Figure 1c).

In summary, the spectral characteristics of *G. biloba* leaves were predominantly driven by phenological stage, with the yellow-leaf stage exhibiting fundamentally altered spectra. Sex-related differences were most prominent at the green-leaf stage, suggesting that this period represents a critical window for sex identification.

### 2.2. Principal Component Analysis (PCA) and Data Distribution Characteristics

**Figure 2:**
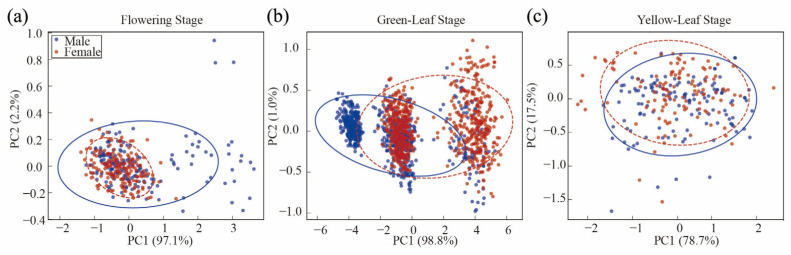
PCA score plots of hyperspectral data from male and female *G*. *biloba* leaves at three phenological stages: (**a**) flowering, (**b**) green-leaf, (**c**) yellow-leaf. Ellipses denote 95% confidence regions. Cumulative variance explained by PC1 and PC2 is indicated in each subplot.

To evaluate the separability of sex-related differences under unsupervised conditions, PCA was applied to the hyperspectral data of each phenological stage, and 95% confidence ellipses were plotted (Figure 2). At the flowering (Figure 2a) and green-leaf stages (Figure 2b), the first two principal components accounted for 99.3% and 99.8% of the cumulative variance, respectively, indicating that nearly all original spectral information was retained after dimensionality reduction. However, the 95% confidence ellipses of male and female samples overlapped substantially at both stages, with only a marginal shift along PC1. At the yellow-leaf stage, the PC1 contribution dropped to 78.7%, and the distributions of the two sexes merged further, becoming visually indistinguishable.

These results indicate that spectral variation is predominantly driven by phenology-related physiological and biochemical factors (e.g., leaf color, water status) shared by both sexes, whereas sex-related spectral differences are weak and represent subordinate variance. As an unsupervised method, PCA preferentially extracts the directions of maximum variance, thereby masking sex-related signals. This does not, however, negate the existence of sex-based spectral differences; it merely demonstrates that they are not the dominant source of variance. Accordingly, supervised classification methods, including partial least squares discriminant analysis (PLS-DA) and linear discriminant analysis (LDA), were introduced in subsequent analyses to leverage class labels for extracting sex-specific spectral features otherwise obscured by the dominant variance.

### 2.3. Stage-Specific Modeling and Algorithm Comparison

**Figure 3:**
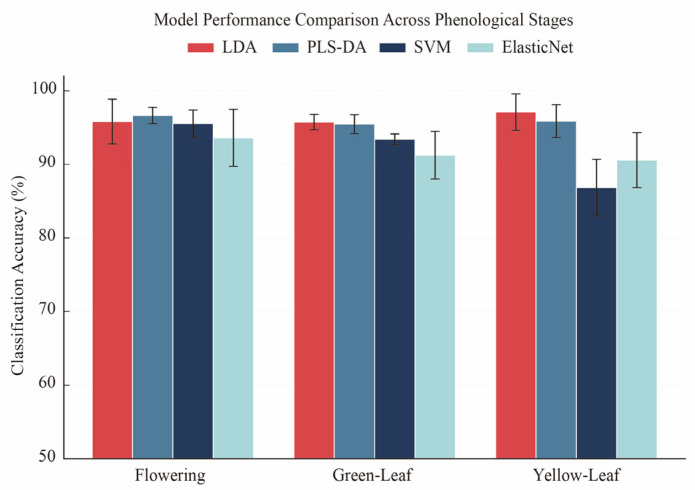
Robustness comparison of different algorithms (LDA, PLS-DA, SVM, Elastic Net) for sex identification of *G. biloba* leaves at flowering, green-leaf, and yellow-leaf stages.

To evaluate the discriminative power of hyperspectral data for sex identification at each phenological stage, independent classification models were constructed for the flowering, green-leaf, and yellow-leaf stages. The optimal model at each stage achieved a test accuracy exceeding 95%, yet the responses to preprocessing methods and classification algorithms varied across stages (Figure 3). At the flowering stage, multiplicative scatter correction (MSC) combined with partial least squares discriminant analysis (PLS-DA) yielded the best performance, with test accuracy, recall, and F1 score all reaching 96.67%. The small gap between training and test accuracy (97.71% vs. 96.67%) indicated favorable generalization. At the green-leaf stage, which had the largest sample size, linear discriminant analysis (LDA) applied directly to raw spectra achieved a test accuracy of 95.77%. Notably, despite exhibiting the most pronounced sex-related reflectance differences, this stage did not produce substantially higher accuracy than the other stages. At the yellow-leaf stage, the combination of MSC and LDA reached a test accuracy of 97.12%, with recall and F1 score of 97.06% and 97.11%, respectively. In addition, the overall confusion matrices, precision, and specificity for all evaluated models are presented in Tables S1–S3. In the cross-algorithm comparison of stability, LDA and PLS-DA demonstrated strong robustness across all three stages, with LDA achieving the optimal results at both the green-leaf and yellow-leaf stages (Figure 3). In contrast, the support vector machine (SVM) algorithm showed considerable performance fluctuation: its accuracy at the yellow-leaf stage was markedly lower than that of the other algorithms, and the associated error bars were larger, indicating weaker generalization ability than the linear models on this dataset.

### 2.4. Key Wavelength Selection and Model Optimization

**Figure 4:**
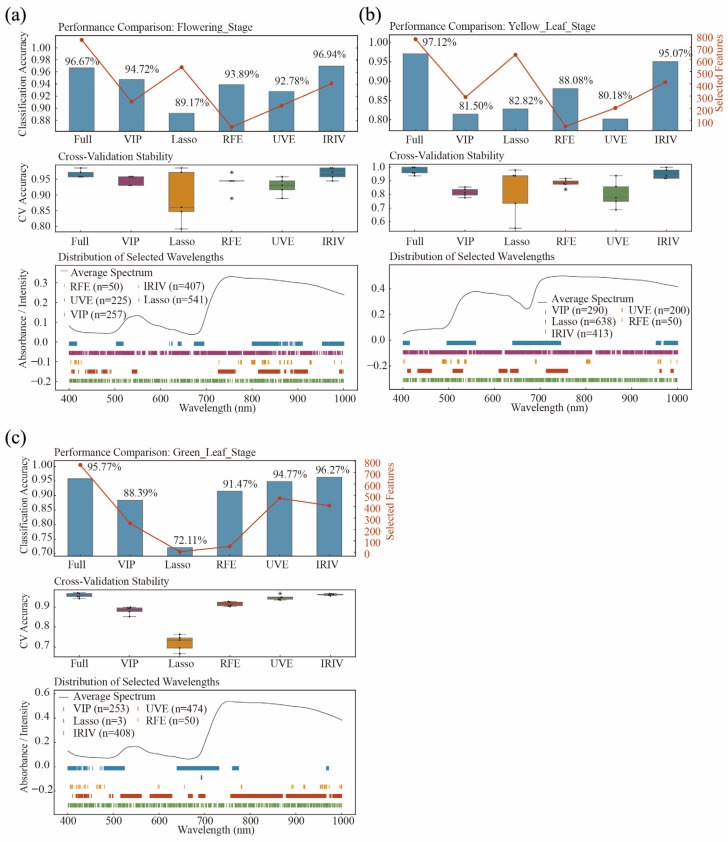
Performance of sex identification models using full-band spectra and feature subsets selected by VIP, Lasso, RFE, UVE, and IRIV. (**a**) Flowering stage, (**b**) yellow-leaf stage, (**c**) green-leaf stage.

To reduce redundancy and identify spectral regions critical for sex identification, five feature selection algorithms—VIP, Lasso, RFE, UVE, and IRIV—were applied at each phenological stage, and the resulting classification performance was evaluated. In terms of accuracy, IRIV achieved the best results at the flowering (Figure 4a) and green-leaf (Figure 4c) stages, with accuracies of 96.94% and 96.27%, slightly exceeding the corresponding full-band models (96.67% and 95.77%). This suggests that the original spectra contained redundancy or noise, and the 400 features retained by IRIV improved model generalization. At the yellow-leaf stage (), the pattern differed: the full-band model led with 97.12% accuracy, while all feature selection methods showed varying declines. IRIV maintained a relatively high accuracy of 95.07%, whereas VIP (81.50%) and UVE (80.18%) dropped markedly. This may be because drastic changes in leaf biochemical composition at the yellow-leaf stage produced more complex spectral distributions, hindering accurate identification of key wavelengths by some algorithms.

Regarding stability, IRIV exhibited consistently small cross-validation standard deviations (±1.62% at flowering, ±0.37% at green-leaf), outperforming the other methods in robustness. Lasso selected only three features at the green-leaf stage, with accuracy plunging to 72.11%, revealing a tendency toward excessive sparsity in high-dimensional spectral data. Although RFE stably controlled feature number, its accuracy remained between 88% and 94%, failing to fully exploit the spectral information. Overall, IRIV achieved a favorable balance between dimensionality reduction and classification accuracy.

To further elucidate the physical basis of spectral sex identification, the top 50 feature wavelengths ranked by weight in the optimal model (LDA or PLS-DA) at each stage were extracted and summarized with a tolerance of ±20 nm (Table 1). The results revealed pronounced phenological differentiation in the spectral features underlying sex identification: the green-leaf and yellow-leaf stages shared no common feature bands, indicating a fundamental shift in the spectral basis for sex identification as leaves senesced. Specifically, the eight feature bands unique to the green-leaf stage were concentrated in the 520–690 nm range, likely associated with spectral differences in chlorophyll and flavonoid absorption. The eight bands unique to the yellow-leaf stage were distributed across 700–1000 nm, potentially reflecting NIR signatures of cellular structural changes and water content during senescence. The flowering stage, as a transitional phase, shared with the green-leaf stage one feature band in the red-edge region (680–700 nm), which is highly sensitive to chlorophyll content, and shared three bands with the yellow-leaf stage (500–520 nm, 840–860 nm, and 980–1000 nm), corresponding to carotenoid absorption, leaf internal scattering, and water absorption, respectively.

### 2.5. Biological Stability of Cross-Stage Spectral Features and Generalization Boundaries

Although stage-specific models had achieved high accuracy, whether stable, phenology-independent spectral sex markers existed in *G. biloba* leaves remained unclear. To address this, a unified classification model was constructed by integrating data from all three stages, using IRIV feature selection with LDA classification. Five-fold cross-validation on the combined dataset yielded an accuracy of 89.24%. When a biological constraint was further imposed by excluding the green-sensitive region (495–570 nm), accuracy dropped by only 0.30%. However, a more stringent test revealed the limitation: a model trained on green-leaf samples and tested on flowering and yellow-leaf samples achieved accuracies of 50.00% and 48.56%, respectively—indistinguishable from random guessing. This sharp decline reveals that the seemingly high internal validation accuracy of the pooled model did not reflect genuine cross-stage generalization. Instead, the model had predominantly learned stage-specific rather than sex-specific spectral features, demonstrating that the spectral basis for sex discrimination is fundamentally phenology-dependent.

**Figure 5:**
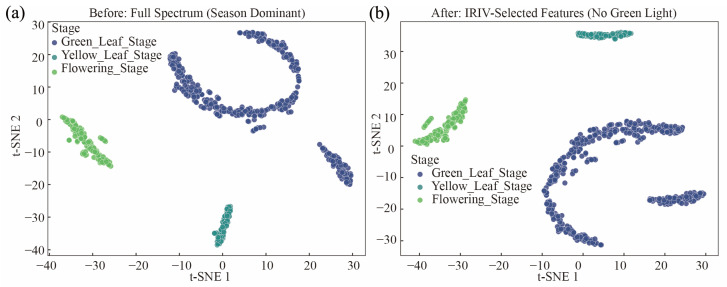
t-SNE visualization of spectral feature space. (**a**) Full-spectrum data. (**b**) IRIV-selected feature subset. Different colors denote different phenological stages.

Visualization further demonstrated that, using both full-spectrum data (Figure 5a) and IRIV-selected features (Figure 5b), samples from the three stages formed distinct clusters in feature space, indicating that dominant spectral variation originated from phenological rather than sex differences. The 89.24% accuracy of the unified model thus reflected the model’s capture of weak within-stage sex signals, rather than learning sex-specific features transferable across stages. This result indicates, conversely, that stage-specific modeling is a necessary consequence of the strong phenological dependency inherent *in G. biloba* spectral sex markers, rather than merely an optional strategy.

### 2.6. Leaf Compound Index Analysis and Flavonoid Content Determination

**Figure 6:**
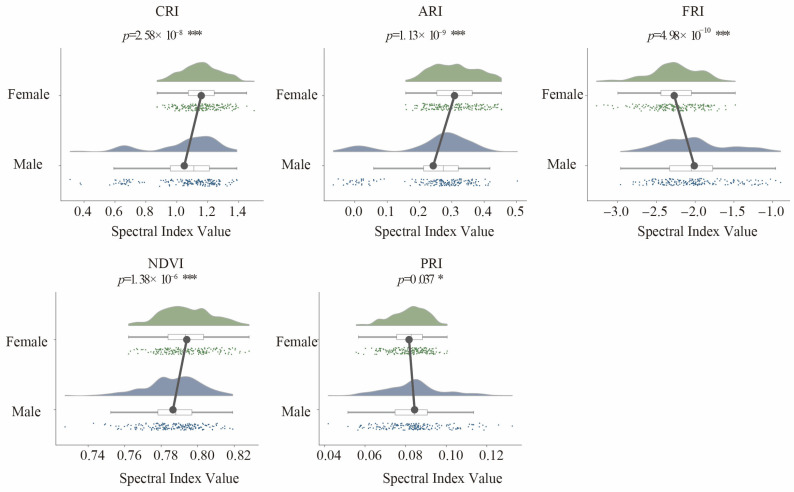
Raincloud plots comparing five spectral indices between male and female *G. biloba* leaves at the flowering stage. (**a**) Carotenoid reflectance index (CRI), (**b**) anthocyanin reflectance index (ARI), (**c**) flavonol reflectance index (FRI), (**d**) normalized difference vegetation index (NDVI), (**e**) photochemical reflectance index (PRI). Each panel displays a raincloud plot comprising a density curve, boxplot, and scatter points. Black dots represent means, connected by a line. Asterisks indicate significant differences (*** *p* < 0.001, * *p* < 0.05 independent-samples *t*-test).

Five spectral indices were calculated from flowering-stage hyperspectral data. All five—carotenoid reflectance index (CRI), anthocyanin reflectance index (ARI), flavonol reflectance index (FRI), normalized difference vegetation index (NDVI), and photochemical reflectance index (PRI)—differed significantly between the sexes (*p* < 0.001). Male trees had higher PRI and FRI, whereas CRI, ARI and NDVI were slightly higher in females, with the two sex groups showing a clear separation in distribution (Figure 6). These results indicate that stable sex-based divergence in pigment accumulation and photosynthetic physiology was already established at the flowering stage.

**Figure 7:**
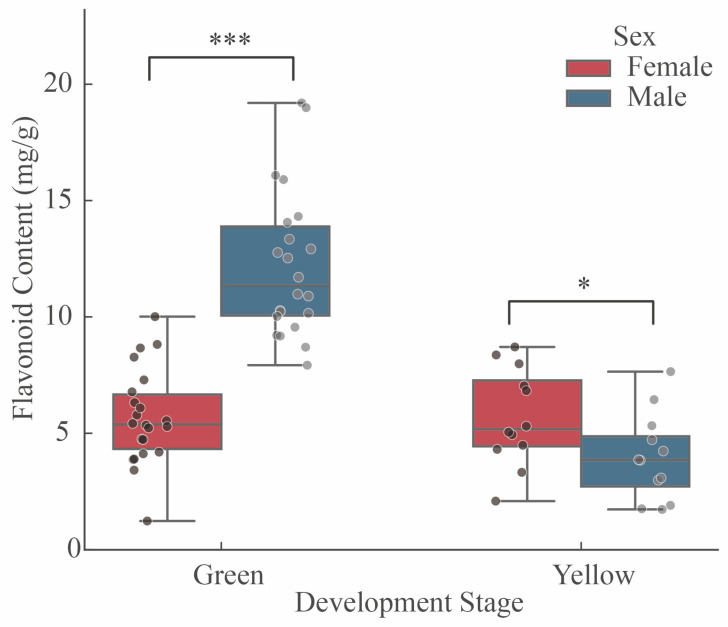
Flavonoid content of male and female *G. biloba* leaves at the green-leaf and yellow-leaf stages, showing the significant stage × sex interaction (*** *p* < 0.001, * *p* < 0.05).

To test whether these biochemical differences remained stable across phenological stages, flavonoid content was measured at the green-leaf and yellow-leaf stages. Two-way ANOVA (Table 2) revealed a highly significant stage × sex interaction (F_1,64_ = 44.56, *p* < 0.001), a significant main effect of sex (F_1,64_ = 78.77, *p* < 0.001), and a non-significant main effect of stage (*p* = 0.984). At the green-leaf stage, males had significantly higher flavonoid content than females (Tukey HSD, *p* < 0.001), with greater inter-individual variation among males. At the yellow-leaf stage, the pattern reversed: male content dropped sharply (mean difference = −8.26, *p* < 0.001), whereas female content remained relatively stable, showing no significant change between stages (*p* = 1.0), which accounted for the non-significant main effect of stage. Although the overall Tukey test did not detect a significant sex difference at the yellow-leaf stage, an independent-samples *t*-test on the yellow-leaf data alone indicated that females had significantly higher flavonoid content than males (t = −2.158, *p* = 0.042). This confirms that the direction of sex-based differences in flavonoid content reversed during senescence (Figure 7).

## 3. Discussion

### 3.1. Within a Single Phenological Stage, Physiological and Biochemical Differences Between Male and Female G. biloba Trees Are Stable and Can Be Effectively Captured by Spectral Techniques

The dominant mechanisms governing leaf reflectance differ fundamentally between the visible and near-infrared (NIR) regions. In the visible region (400–700 nm), reflectance is primarily determined by the absorption characteristics of photosynthetic pigments: chlorophyll exhibits strong absorption bands near 670 nm (red) and 450 nm (blue), with the weakest absorption near 550 nm (green), giving rise to the characteristic green peak and red valley [27]. In the NIR region (700–1300 nm), reflectance is largely independent of pigment absorption and is instead dominated by multiple scattering at cell–air interfaces within the leaf internal structure, particularly in the spongy mesophyll [18,19].

Consistent with these spectral principles, stable and interpretable sex-related spectral differences were observed at each phenological stage. At the green-leaf stage, discriminative bands were concentrated in the 520–690 nm range, which covers the absorption domains of flavonoid (520–650 nm) and the red-light absorption region of chlorophyll (650–690 nm) [25], suggesting that sex-related spectral differences at this stage primarily originate from divergence in photosynthetic and secondary pigment contents between male and female trees. This inference aligns with previous reports of significant sex-based differences in chlorophyll content in *G. biloba* [29]. At the yellow-leaf stage, as chlorophyll rapidly degraded while carotenoids were relatively retained, pigment absorption features in the visible region weakened overall, and the spectral features for sex identification shifted to the NIR region (700–1000 nm). These bands covered the NIR scattering plateau (890–910 nm) and the edge of water absorption bands (930–970 nm) [26], corresponding to differences in leaf internal cellular structure and water status, respectively. This spectral shift is consistent with asynchronous senescence between male and female *G. biloba* trees, indicating potential sex-based differences in senescence rate, cellular structural degradation, and water retention capacity [21,29,30].

The flowering-stage spectral index analysis further confirmed the stability of sex-based differentiation within a single phenological stage. All five spectral indices calculated from flowering-stage hyperspectral data—CRI, ARI, FRI, NDVI, and PRI—showed significant sex differences (*p* < 0.05). Among these, NDVI reflects vegetation greenness and chlorophyll content [31,32], and PRI is sensitive to photosynthetic efficiency and light-stress status [33]; both indices are associated with photosynthetic capacity, and sex-related differences in photosynthetic performance have been previously documented in *G. biloba* [30,34]. These spectral index results, together with the high accuracy (>95%) achieved by stage-specific models, mutually corroborate the conclusion that physiological and biochemical differences between male and female *G. biloba* trees exist stably within a given phenological stage and can be effectively captured by hyperspectral techniques.

### 3.2. Limitations in Cross-Stage Generalization of Sex Identification Models for Mature G. biloba

Chen et al. adopted a stage-pre-discrimination strategy by constructing separate models for the green-leaf and yellow-leaf stages, first demonstrating the feasibility of hyperspectral imaging for sexing mature *G. biloba* trees [22]. To test whether a universal cross-stage model was equally feasible, data from all three phenological stages were combined, yielding 89.24% accuracy under internal cross-validation, comparable to the 87.74% reported for pooled two-stage data [22]. However, when a model trained on one stage was directly applied to another, accuracy dropped to approximately 50%—the level of random guessing. This suggests that the seemingly high internal validation accuracy of the combined model did not reflect learning of sex-specific features. t-SNE analysis revealed the underlying cause: samples from the three stages formed distinct clusters in feature space, with phenological differences dominating spectral variation and thereby masking sex-related signals. The combined model therefore captured phenological rather than sex-related information. Feature band analysis provided independent evidence: with a ±20 nm tolerance window, the green-leaf and yellow-leaf stages shared no common discriminative bands, while the flowering stage, as a transitional phase, shared only one band with the green-leaf stage and three bands with the yellow-leaf stage (Table 1). Thus, almost no discriminative bands were shared across all three phenological stages, confirming a fundamental shift in spectral sex markers with phenology. This complete failure of cross-stage generalization constitutes the most compelling evidence that the spectral basis for sex discrimination is fundamentally phenology-dependent, and that stage-specific models are not merely a methodological preference but a necessary strategy for practical sex identification in *G. biloba*.

In dioecious plants, asymmetric reproductive investment can drive sexual divergence in physiological traits such as photosynthesis [14] and antioxidant capacity [15], reflected in leaf pigments and secondary metabolites, which may vary with phenology. Such phenology-dependent shifts in sex-related traits have been reported for chlorophyll indices in poplar [16], and in *G. biloba*, Ye et al. (2022) found that sex-based differences in total flavonoid content changed over time [30]. These findings indicate that physiological and biochemical differences between sexes are not fixed markers but change dynamically across phenological stages. Because leaf spectral features fundamentally reflect pigment composition, secondary metabolites, and water status, phenological instability in these traits necessarily leads to instability in spectral sex markers, implying that sex-related spectral differences in *G. biloba* may also shift across stages.

### 3.3. Limitations and Future Directions

This study has several limitations. First, all samples were collected from a single site, and the sample sizes at the flowering and yellow-leaf stages were relatively limited. Although the permanently marked experimental population provided a valuable site-level biological control, single-site sampling inherently limits generalizability to other regions, climates, or soil environments. Therefore, cross-regional, cross-cultivar, and multi-year validation with larger and more balanced sample sizes is required before practical deployment. A related concern pertains specifically to the yellow-leaf stage, where the number of trees was substantially smaller than at the other two stages (eight per sex). This reduction resulted from a combination of a narrow senescence window, unexpected strong winds that accelerated leaf fall on the scheduled sampling day, and a strict plant health quality-control protocol that excluded any individuals showing signs of disease or physiological stress. Although the male and female sample sizes were well balanced within each phenological stage, the overall sample sizes across stages remained substantially imbalanced, and no explicit sample balancing was applied to address this. Future studies should therefore adopt sample balancing techniques or leave-one-phenological-stage-out cross-validation to obtain more robust and unbiased estimates, and should also seek to increase the number of trees sampled at the yellow-leaf stage.

Second, within each phenological stage, the training and test sets were split at the leaf level rather than at the tree level. Leaves from the same tree share certain spectral similarities owing to their common genetic background and micro-environment. Consequently, this splitting strategy may cause leaves from the same individual to appear in both sets, potentially inflating the classification accuracy of the stage-specific models to some degree. To obtain more conservative and unbiased estimates of model generalization performance, future studies should adopt a tree-level splitting strategy, in which all leaves from a given tree are assigned exclusively to either the training set or the test set.

Third, biochemical validation was limited to flavonoid content. It is tentatively inferred that the visible bands may be attributable to chlorophyll absorption and that the near-infrared bands may reflect cellular structure and water status, based on known spectral properties and the existing literature. However, direct measurements of these constituents were not performed in the present study. Future work should incorporate quantitative analyses of chlorophylls, carotenoids, anthocyanins, and water content, together with leaf anatomical observations, to establish a more complete and directly validated spectrum–physiology relationship.

Fourth, although the ±20 nm tolerance window analysis provided reasonable evidence that discriminative bands shift with phenology, formal statistical testing or stability screening procedures are still needed to further validate the degree of band overlap across stages.

In addition to the methodological limitations discussed above, practical considerations also merit attention. From a practical standpoint, the high cost of hyperspectral imaging equipment may hinder widespread adoption. Future work could explore transferring the key discriminative bands (e.g., 520–690 nm and 700–1000 nm) to multispectral or portable near-infrared devices, thereby lowering the technological barrier. Furthermore, this study focused on mature *G. biloba* trees, in which sex-related physiological differentiation has become relatively stable. Future research should evaluate the applicability of the present methodological framework to juvenile trees, aiming to enable sex prediction at an earlier developmental stage.

## 4. Conclusions

This study investigated the phenological stability of spectral sex markers in mature *G. biloba* using hyperspectral imaging. Although the universal model achieved 89.24% accuracy under internal cross-validation, a model trained exclusively on green-leaf samples dropped to near-chance levels when tested on the other two stages. This contrast demonstrates that the models predominantly learned phenological rather than sex-specific information. Furthermore, as leaves senesced, key spectral bands migrated from the visible to the near-infrared region, and flavonoid-based sex differences reversed direction between the green-leaf and yellow-leaf stages. These convergent lines of evidence demonstrate that spectral sex markers in *G. biloba* are fundamentally phenology-dependent: when data are pooled across stages, phenological variation overwhelms sex-related signals. Consequently, stage-specific modeling is not a methodological preference but a necessary strategy dictated by the biology of the system. Hence, future hyperspectral-based sex identification workflows for *G. biloba* should prioritize classifiers tailored to specific phenological stages.

## 5. Materials and Methods

### 5.1. Plant Materials

All leaf samples were collected from a permanently marked experimental population of over 400 *G. biloba* trees of known sex at the Yuquanshan nursery in Haidian District, Beijing. These trees were mature individuals approximately 25–30 years old. Sampling was conducted at three phenological stages: flowering (March 2025; 100 trees per sex, 3 leaves per tree), green-leaf (July 2025; 150 trees per sex, 9 leaves per tree), and yellow-leaf (November 2025; 8 trees per sex, 243 leaves total). After screening, low-quality spectra were removed, yielding the following final sample sizes for modeling: green-leaf stage, 675 female and 720 male leaves; flowering stage, 180 leaves per sex; yellow-leaf stage, 118 female and 125 male leaves.

### 5.2. Methods

#### Hyperspectral Imaging of *G. biloba* Leaves and Reflectance Extraction

The hyperspectral imaging system consisted of the following components: a V10E linear-scanning imaging spectrograph (Spectral Imaging Ltd., Oulu, Finland); a 1004 × 1002 pixel CCD camera, model EM285CL (Raptor Photonics Ltd., Belfast, Northern Ireland, UK); two 150 W halogen lamps, model IT 3900 e (Illumination Technologies Inc., New York, NY, USA); and a motorized translation stage, model IRCP0076-1COMB, with corresponding spectral image acquisition software (both provided by Wuling Optical Co., Ltd., Hsinchu, Taiwan, China). The entire system was housed in a custom-built light-tight dark box to eliminate ambient stray light during spectral acquisition.

All hyperspectral images were acquired in 2025. To eliminate ambient stray light, the entire imaging process was conducted in a light-tight chamber. Prior to scanning, system parameters were optimized based on preliminary experiments: the motorized translation stage was set to a speed of 1.7 mm/s, and the exposure time of the imaging unit was set to 6.5 ms to ensure image sharpness without overexposure.

Leaves were spread flat on the moving stage and scanned in push-broom mode across the full spectral range, generating raw hyperspectral data cubes covering the entire leaf surface.

Owing to non-uniform illumination and camera dark current, raw images contained noise. Before spectral extraction, reflectance calibration was performed using HSI Analyzer software (Wuling Optical Co., Ltd., Taiwan, China). A white reference image W was acquired from a standard diffuse reflectance whiteboard (reflectance ≈ 100%), and a dark reference image B was acquired with the light source turned off and the lens cap on (reflectance ≈ 0%). The calibrated image I was calculated according to Equation (1):

(1) $$I=\left(I_{0}-B\right)/\left(W-B\right)$$

where I_0_ denotes the raw hyperspectral image.

After calibration, a dynamic threshold segmentation algorithm was applied to remove the background and precisely isolate the leaf region. Morphological filtering and masking were then employed to further suppress edge noise, yielding the final region of interest (ROI). Considering that the spectral bands at both ends of the range are strongly affected by instrumental noise and exhibit low signal-to-noise ratios, reflectance data from 400 to 1000 nm (comprising 766 bands) were retained for subsequent analysis. This spectral range was divided into a visible (VIS) region (400–780 nm, 490 bands) and a near-infrared (NIR) region (780–1000 nm, 276 bands).

### 5.3. Data Processing

The data processing workflow in this study comprised dimensionality reduction and visualization of hyperspectral data, spectral preprocessing, characteristic wavelength selection, construction of discriminant models, and model robustness validation. All analyses were performed in Python 3.1.4 using machine learning libraries including Scikit-learn (version 1.3.0) and LightGBM (version 4.0.0).

#### 5.3.1. Principal Component Analysis

Principal component analysis (PCA) was applied to the raw spectral data for dimensionality reduction and visualization. Score plots of the first two or three principal components were used to examine the natural grouping and separation of different sample classes and to identify potential outliers.

#### 5.3.2. Spectral Preprocessing

Raw hyperspectral data typically contain interference from light scattering, baseline drift, and random noise. To improve the signal-to-noise ratio, several preprocessing algorithms were compared, including standard normal variate (SNV), multiplicative scatter correction (MSC), and Savitzky–Golay smoothing derivatives (first-derivative SG-1D and second-derivative SG-2D). SNV and MSC primarily correct for scattering effects and optical path length variations, whereas SG derivatives resolve baseline drift and enhance subtle absorption features. The optimal preprocessing method was selected based on classification accuracy for subsequent analysis.

#### 5.3.3. Spectral Preprocessing, Model Construction, and Evaluation

Multiplicative scatter correction (MSC) was applied to correct for scattering effects and optical path length variations, with raw spectra retained as a control to assess preprocessing impacts.

Based on both full-spectrum and characteristic-wavelength data, four supervised classification models were constructed to discriminate sample classes: linear discriminant analysis (LDA), partial least squares discriminant analysis (PLS-DA), support vector machine (SVM), and elastic net (Elastic Net). All models were evaluated using stratified K-fold cross-validation (K = 5) with a fixed global random seed (random_state = 42). The dataset was randomly partitioned into five folds while preserving the class distribution in each fold. Model performance was evaluated using accuracy, F1 score, recall, precision, and specificity.

#### 5.3.4. Characteristic Wavelength Selection

Full-spectrum hyperspectral data exhibit high collinearity and contain substantial redundant variables that impair model efficiency. To identify the most discriminative wavelengths, five feature selection methods were employed: uninformative variable elimination (UVE), variable importance in projection (VIP), the least absolute shrinkage and selection operator (Lasso), recursive feature elimination (RFE), and iteratively retaining informative variables (IRIV). The feature subset achieving the highest classification accuracy was retained for model construction.

All wavelength selection algorithms were strictly nested within the cross-validation procedure. In each fold, feature ranking and selection were performed exclusively on the training subset, while the held-out validation fold was never exposed to the selection process, preventing any leakage of test-set information into the feature selection step. The feature subset achieving the highest classification accuracy was retained for model construction.

#### 5.3.5. Leaf Compound Index Analysis

Spectral reflectance is correlated with the content of specific leaf compounds, allowing their estimation through dedicated spectral indices. Accordingly, hyperspectral reflectance of ginkgo leaves was measured and the following indices were calculated: anthocyanin reflectance index (ARI) [35], flavonol reflectance index (FRI) [36], carotenoid reflectance index (CRI) [36], normalized difference vegetation index (NDVI) [37], and photochemical reflectance index (PRI) [33]. Differences in these indices between male and female trees were then examined. The indices were calculated as follows:

(2) $$ARI=R_{800}\left(1/R_{550}-1/R_{700}\right)$$

(3) $$FRI=R_{800}\left(1/R_{410}-1/R_{460}\right)$$

(4) $$CRI=R_{800}\left(1/R_{520}-1/R_{700}\right)$$

(5) $$NDVI=\left(R_{800}-R_{670}\right)/\left(R_{800}+R_{670}\right)$$

(6) $$PRI=\left(R_{531}-R_{570}\right)/\left(R_{531}+R_{570}\right)$$

#### 5.3.6. Determination of Flavonoid Content in *G. biloba* Leaves

Flavonoid content was measured at the green-leaf and yellow-leaf stages. Leaf samples were oven-dried to constant weight, ground, and sieved through a 30–50 mesh screen, after which approximately 0.1 g of each sample was weighed. Flavonoid content was determined using a spectrophotometer (model UV-1600PC, Shanghai Mapada Instruments Co., Ltd., Shanghai, China) and a plant flavonoid assay kit (Solarbio Science & Technology Co., Ltd., Beijing, China).

## Figures and Tables

**Table 1:** Classification of Characteristic Bands and Spectral Significance at Different Phenological Stages (±20 nm Tolerance).

Band Category	Number of Bands	Characteristic Band Intervals (nm)	Probable Corresponding Spectral Features
Flowering	3	400–420, 820–840, 830–850	Carotenoid absorption [23]; leaf internal structure reflectance [19]
Green-leaf	8	520–540, 570–590, 620–640, 630–650, 640–660, 650–670, 660–680, 670–690	Flavonoid absorption [24]; chlorophyll a/b absorption [25]
Yellow-leaf	8	700–720, 890–910, 930–950, 940–960, 950–970, 960–980, 970–990, 980–1000	Leaf internal structure [19]; water content [26]
Flowering & Green-leaf	1	680–700	Red-edge band, sensitive to chlorophyll [27], related to photosynthetic function [28]
Flowering & Yellow-leaf	3	500–520, 840–860, 980–1000	Carotenoid [23] and flavonoid absorption [24]; leaf structural reflectance [19]; water absorption [26]
All three stages	0	—	—

**Table 2:** Results of two-way analysis of variance for flavonoid content in *G. biloba* leaves.

Source	df	Mean Square	F	*p*
Stage	1	0.00	0.00	0.984
Sex	1	470.54	78.77	<0.001
Stage × Sex	1	266.17	44.56	<0.001
Error	64	5.97	-	-

## Data Availability

The data presented in this study are available on reasonable request from the corresponding author.

## Supplementary Materials

The following supporting information can be downloaded at: https://www.mdpi.com/article/10.3390/plants15152255/s1, Table S1. Confusion matrix-derived evaluation metrics for all models at the green-leaf stage. Table S2. Confusion matrix-derived evaluation metrics for all models at the yellow leaf stage. Table S3. Confusion matrix-derived evaluation metrics for all models at the at the flowering stage.
