# Supplementary material for: Phenology-Dependent Sex Identification in Mature Ginkgo biloba Using Hyperspectral Imaging
Source: Plants (Basel). 2026 Jul 23;15(15):2255. doi: 10.3390/plants15152255 (PMC13468224; doi:10.3390/plants15152255)
Supplement: Supplementary file 1 [file plants-15-02255-s001.zip › plants-4405223-supplementary.pdf]

Table S1. Confusion matrix-derived evaluation metrics for all models at the green-leaf stage.

Model	Run_ID	Precision_Class_0	Precision_Class_1	Specificity_Class_0	Specificity_Class_1	TP_Class_0_Int	FN_Class_0_Int	FP_Class_0_Int	TP_Class_1_Int
LDA	None ∣ LDA	0.9611	0.9546	0.9639	0.9511	642	33	26	694
PLS-DA	SG-1D ∣ PLS-DA	0.95	0.9594	0.9528	0.957	646	29	34	686
PLS-DA	SNV ∣ PLS-DA	0.9411	0.9497	0.9444	0.9467	639	36	40	680
PLS-DA	MSC ∣ PLS-DA	0.9423	0.9471	0.9458	0.9437	637	38	39	681
SVM	SG-1D ∣ SVM	0.9158	0.9524	0.9181	0.9511	642	33	59	661
PLS-DA	None ∣ PLS-DA	0.938	0.9251	0.9431	0.9185	620	55	41	679
PLS-DA	SG-2D ∣ PLS-DA	0.9242	0.9266	0.9292	0.9215	622	53	51	669
LDA	SG-1D ∣ LDA	0.9287	0.9144	0.9347	0.9067	612	63	47	673
ElasticNet	SG-1D ∣ ElasticNet	0.9048	0.9199	0.9097	0.9156	618	57	65	655
SVM	SG-2D ∣ SVM	0.8671	0.9368	0.8653	0.9378	633	42	97	623
MLP	SG-1D ∣ MLP	0.8941	0.9063	0.9	0.9007	608	67	72	648
ElasticNet	SG-2D ∣ ElasticNet	0.8927	0.895	0.9	0.8874	599	76	72	648
LDA	MSC ∣ LDA	0.8824	0.9051	0.8875	0.9007	608	67	81	639
LDA	SNV ∣ LDA	0.8799	0.9048	0.8847	0.9007	608	67	83	637
LightGBM	SG-1D ∣ LightGBM	0.8785	0.8947	0.8847	0.8889	600	75	83	637
LightGBM	MSC ∣ LightGBM	0.8852	0.8881	0.8931	0.88	594	81	77	643
LDA	SG-2D ∣ LDA	0.8872	0.8742	0.8972	0.8622	582	93	74	646
ElasticNet	SNV ∣ ElasticNet	0.8667	0.8908	0.8722	0.8859	598	77	92	628
LightGBM	SG-2D ∣ LightGBM	0.8677	0.8897	0.8736	0.8844	597	78	91	629
MLP	SG-2D ∣ MLP	0.8529	0.9031	0.8542	0.9022	609	66	105	615
LightGBM	SNV ∣ LightGBM	0.8792	0.8731	0.8889	0.8622	582	93	80	640
ElasticNet	MSC ∣ ElasticNet	0.8644	0.8843	0.8708	0.8785	593	82	93	627
MLP	MSC ∣ MLP	0.8686	0.8275	0.8861	0.803	542	133	82	638
MLP	SNV ∣ MLP	0.8363	0.8494	0.8458	0.84	567	108	111	609
SVM	None ∣ SVM	0.8407	0.8302	0.8556	0.8133	549	126	104	616
SVM	SNV ∣ SVM	0.8279	0.8377	0.8389	0.8267	558	117	116	604
SVM	MSC ∣ SVM	0.8267	0.8375	0.8375	0.8267	558	117	117	603
LightGBM	None ∣ LightGBM	0.813	0.8282	0.8236	0.8178	552	123	127	593

MLP	None ∣ MLP	0.7899	0.7847	0.8097	0.763	515	160	137	583
ElasticNet	None ∣ ElasticNet	0.7275	0.7331	0.7514	0.7081	478	197	179	541

Note: Class_0 = female; Class_1 = male.

Run_ID denotes the combination of spectral preprocessing and classification algorithms.

The confusion matrix components (TP = true positives, FN = false negatives, FP = false positives) and the integer counts (_Int) represent the pooled validation results accumulated across all 5 cross-validation folds.

For binary classification, True Negatives (TN) for Class_0 correspond to the TP of Class_1. Precision = TP / (TP + FP); Specificity = TN / (TN + FP).

Class_0 = female; Class_1 = male.

Table S2. Confusion matrix-derived evaluation metrics for all models at the yellow-leaf stage.

Model	Run_ID	Precision_Class_0	Precision_Class_1	Specificity_Class_0	Specificity_Class_1	TP_Class_0_Int	FN_Class_0_Int	FP_Class_0_Int	TP_Class_1_Int
LDA	MSC ∣ LDA	0.9912	0.9538	0.992	0.9492	112	6	1	124
LDA	SNV ∣ LDA	0.9911	0.9466	0.992	0.9407	111	7	1	124
PLS-DA	MSC ∣ PLS-DA	0.9655	0.9528	0.968	0.9492	112	6	4	121
LDA	None ∣ LDA	0.9655	0.9528	0.968	0.9492	112	6	4	121
PLS-DA	SNV ∣ PLS-DA	0.9573	0.9524	0.96	0.9492	112	6	5	120
PLS-DA	None ∣ PLS-DA	0.9569	0.9449	0.96	0.9407	111	7	5	120
LDA	SG-1D ∣ LDA	0.9565	0.9375	0.96	0.9322	110	8	5	120
PLS-DA	SG-1D ∣ PLS-DA	0.9397	0.9291	0.944	0.9237	109	9	7	118
PLS-DA	SG-2D ∣ PLS-DA	0.9286	0.8931	0.936	0.8814	104	14	8	117
ElasticNet	SG-1D ∣ ElasticNet	0.906	0.9048	0.912	0.8983	106	12	11	114
LDA	SG-2D ∣ LDA	0.9052	0.8976	0.912	0.8898	105	13	11	114

ElasticNet	SG-2D | ElasticNet	0.8772	0.8605	0.888	0.8475	100	18	14	111
SVM	SG-1D | SVM	0.8839	0.855	0.896	0.839	99	19	13	112
SVM	SG-2D | SVM	0.8879	0.8309	0.904	0.8051	95	23	12	113
LightGBM	SG-2D | LightGBM	0.8496	0.8308	0.864	0.8136	96	22	17	108
LightGBM	SG-1D | LightGBM	0.8291	0.8333	0.84	0.822	97	21	20	105
SVM	None | SVM	0.8205	0.8254	0.832	0.8136	96	22	21	104
MLP	SG-1D | MLP	0.8364	0.8045	0.856	0.7797	92	26	18	107
MLP	SG-2D | MLP	0.8273	0.797	0.848	0.7712	91	27	19	106
ElasticNet	MSC | ElasticNet	0.7724	0.8083	0.776	0.8051	95	23	28	97
ElasticNet	SNV | ElasticNet	0.7627	0.776	0.776	0.7627	90	28	28	97
ElasticNet	None | ElasticNet	0.7565	0.7578	0.776	0.7373	87	31	28	97
SVM	MSC | SVM	0.7723	0.7183	0.816	0.661	78	40	23	102
SVM	SNV | SVM	0.7723	0.7183	0.816	0.661	78	40	23	102
LightGBM	MSC | LightGBM	0.75	0.6993	0.8	0.6356	75	43	25	100
MLP	None | MLP	0.7333	0.7029	0.776	0.6525	77	41	28	97
LightGBM	None | LightGBM	0.7117	0.7045	0.744	0.6695	79	39	32	93
LightGBM	SNV | LightGBM	0.708	0.7077	0.736	0.678	80	38	33	92
MLP	MSC | MLP	0.7037	0.6889	0.744	0.6441	76	42	32	93
MLP	SNV | MLP	0.6417	0.6667	0.656	0.6525	77	41	43	82

Note: Class_0 = female; Class_1 = male.

Run_ID denotes the combination of spectral preprocessing and classification algorithms.

The confusion matrix components (TP = true positives, FN = false negatives, FP = false positives) and the integer counts (_Int) represent the pooled validation results accumulated across all 5 cross-validation folds.

For binary classification, True Negatives (TN) for Class_0 correspond to the TP of Class_1. Precision = TP / (TP + FP); Specificity = TN / (TN + FP).

Class_0 = female; Class_1 = male.

Table S3. Confusion matrix-derived evaluation metrics for all models at the at the flowering stage.

Model	Run_ID	Precision_Class_0	Precision_Class_1	Specificity_Class_0	Specificity_Class_1	TP_Class_0_Int	FN_Class_0_Int	FP_Class_0_Int	TP_Class_1_Int
PLS-DA	MSC | PLS-DA	0.9667	0.9667	0.9667	0.9667	174	6	6	174
LDA	MSC | LDA	0.9365	0.9825	0.9333	0.9833	177	3	12	168
LDA	SNV | LDA	0.9365	0.9825	0.9333	0.9833	177	3	12	168
SVM	SG-1D | SVM	0.9607	0.9505	0.9611	0.95	171	9	7	173
PLS-DA	SG-1D | PLS-DA	0.9657	0.9405	0.9667	0.9389	169	11	6	174
PLS-DA	SG-2D | PLS-DA	0.9341	0.9438	0.9333	0.9444	170	10	12	168
PLS-DA	SNV | PLS-DA	0.9341	0.9438	0.9333	0.9444	170	10	12	168
ElasticNet	SG-1D | ElasticNet	0.9538	0.9198	0.9556	0.9167	165	15	8	172
PLS-DA	None | PLS-DA	0.9278	0.9278	0.9278	0.9278	167	13	13	167
LDA	SG-2D | LDA	0.9032	0.931	0.9	0.9333	168	12	18	162
LDA	None | LDA	0.8984	0.9306	0.8944	0.9333	168	12	19	161
ElasticNet	SG-2D | ElasticNet	0.9006	0.905	0.9	0.9056	163	17	18	162
LDA	SG-1D | LDA	0.8962	0.9096	0.8944	0.9111	164	16	19	161
LightGBM	SG-1D | LightGBM	0.8913	0.9091	0.8889	0.9111	164	16	20	160
SVM	SG-2D | SVM	0.8977	0.8804	0.9	0.8778	158	22	18	162
LightGBM	SG-2D | LightGBM	0.8833	0.8833	0.8833	0.8833	159	21	21	159
ElasticNet	MSC | ElasticNet	0.8579	0.9	0.85	0.9056	163	17	27	153
ElasticNet	SNV | ElasticNet	0.8689	0.8814	0.8667	0.8833	159	21	24	156
MLP	SG-1D | MLP	0.8701	0.8579	0.8722	0.8556	154	26	23	157

MLP	SG-2D ∣ MLP	0.8728	0.8449	0.8778	0.8389	151	29	22	158
SVM	SNV ∣ SVM	0.8606	0.8051	0.8722	0.7889	142	38	23	157
SVM	MSC ∣ SVM	0.8545	0.8	0.8667	0.7833	141	39	24	156
LightGBM	MSC ∣ LightGBM	0.8229	0.8054	0.8278	0.8	144	36	31	149
MLP	MSC ∣ MLP	0.8333	0.7917	0.8444	0.7778	140	40	28	152
LightGBM	SNV ∣ LightGBM	0.8274	0.7865	0.8389	0.7722	139	41	29	151
MLP	SNV ∣ MLP	0.8483	0.7349	0.8778	0.6833	123	57	22	158
SVM	None ∣ SVM	0.8082	0.7103	0.8444	0.6556	118	62	28	152
ElasticNet	None ∣ ElasticNet	0.7456	0.7173	0.7611	0.7	126	54	43	137
MLP	None ∣ MLP	0.6436	0.6835	0.6	0.7222	130	50	72	108
LightGBM	None ∣ LightGBM	0.6364	0.6474	0.6222	0.6611	119	61	68	112

Note: Class_0 = female; Class_1 = male.

Run_ID denotes the combination of spectral preprocessing and classification algorithms.

The confusion matrix components (TP = true positives, FN = false negatives, FP = false positives) and the integer counts (_Int) represent the pooled validation results accumulated across all 5 cross-validation folds.

For binary classification, True Negatives (TN) for Class_0 correspond to the TP of Class_1. Precision = TP / (TP + FP); Specificity = TN / (TN + FP).

Class_0 = female; Class_1 = male.
